# Supplementary material for: Integrated multi-omics analysis of adverse cardiac remodeling and metabolic inflexibility upon ErbB2 and ERRα deficiency
Source: Commun Biol. 2022 Sep 12;5:955. doi: 10.1038/s42003-022-03942-4 (PMC9467976; doi:10.1038/s42003-022-03942-4)
Supplement: Supplementary file 2 — Supplementary Information Files [file 42003_2022_3942_MOESM2_ESM.pdf]

Integrated multi-omics analysis of adverse cardiac remodeling and metabolic inflexibility upon ErbB2 and ERRα deficiency

Catherine R. Dufour, Hui Xia, Wafa B'chir, Marie-Claude Perry, Uros Kuzmanov, Anastasiia Gainullina, Kurt Dejgaard, Charlotte Scholtes, Carlo Ouellet, Dongmei Zuo, Virginie Sanguin-Gendreau, Christina Guluzian, Harvey W. Smith, William J. Muller, Etienne Audet-Walsh, Alexey A. Sergushichev, Andrew Emili, Vincent Giguère

Supplementary Information

Supplementary Figures 1-8

Supplementary Figure 1

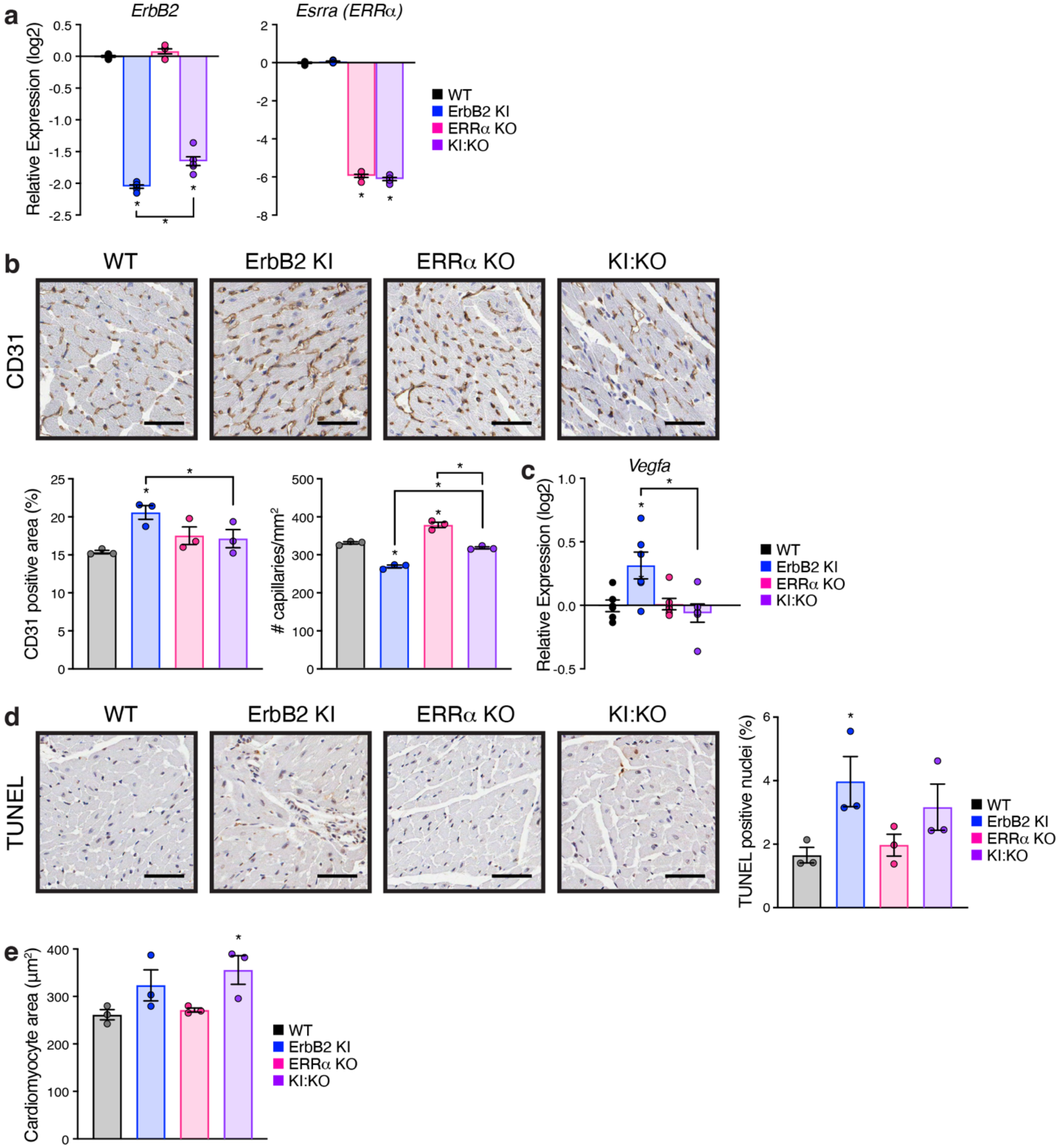

Supplementary Fig. 1 | Histological examination of mouse hearts. **a**, Cardiac qRT-PCR analysis of the ErbB2- and ERRα-encoding genes normalized to *Rplp0* levels (n=6). **b**, Representative staining of mouse heart sections with endothelial marker, CD31, for capillary density assessment. Quantification of CD31 immunoreactivity and capillary number are shown (n=3). Scale bar, 50 μm. **c**, Cardiac qRT-PCR analysis of *Vegfa* expression normalized to *Rplp0* levels (n=6). **d**, Representative TUNEL staining of heart sections for cardiomyocyte apoptosis. Quantification of TUNEL-positive nuclei is shown (n=3). Scale bar, 50 μm. **e**, Quantification of cardiomyocyte diameters (n=3) from WGA-stained mouse heart sections shown in Fig. 1a. Data in **a-e** represent means ±SEM, *p < 0.05 by ANOVA relative to WT controls, unless otherwise indicated. See also Fig. 1.

Supplementary Figure 2

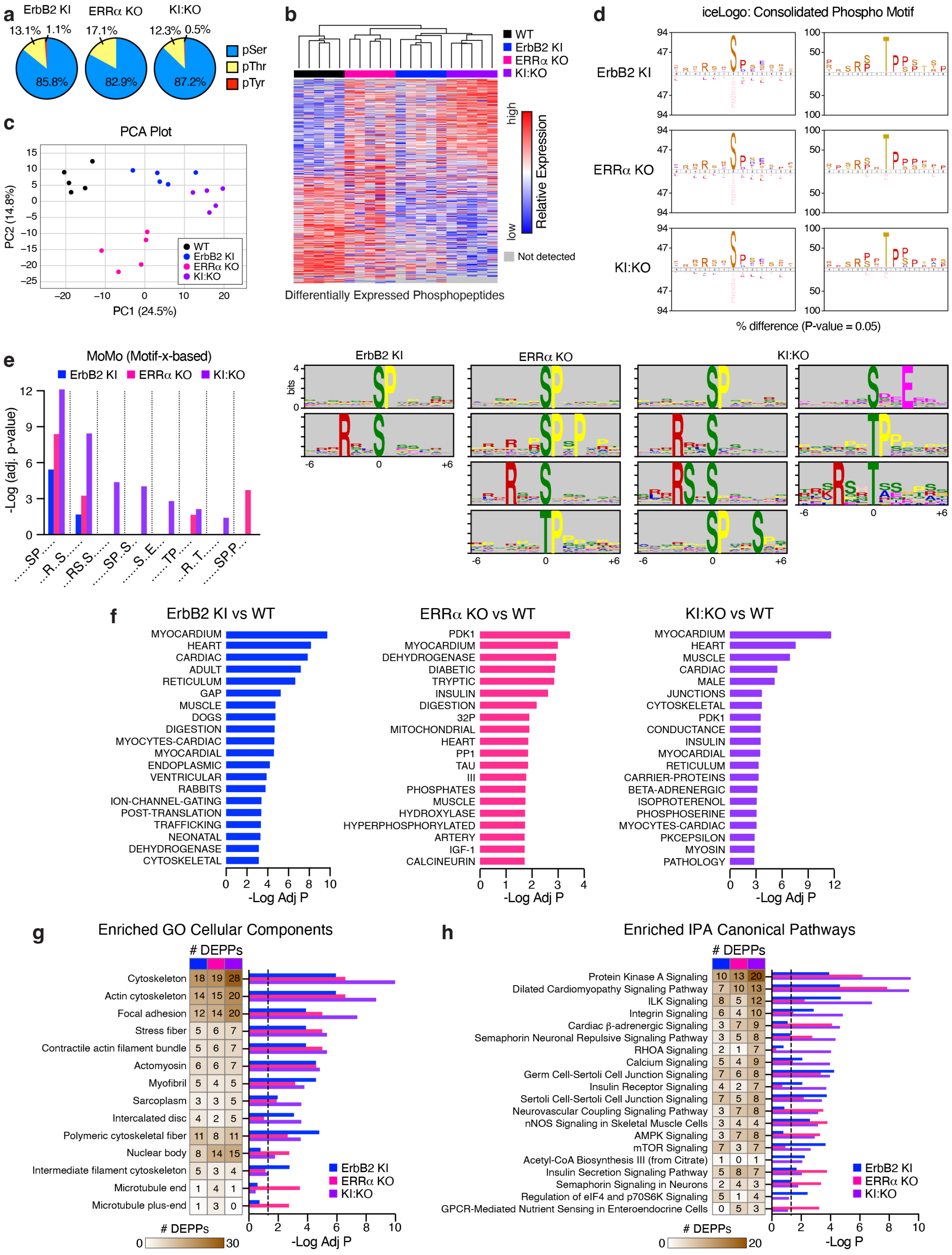

Supplementary Fig. 2 | Phosphoproteomics analysis of mouse hearts lacking ERRα and/or ErbB2 signaling. **a**, Pie charts representing the distribution of phosphorylated serine (pSer), threonine (pThr) and tyrosine (pTyr) residues identified by phosphoproteomics profiling in the mouse models relative to WT controls (limma, $p < 0.05$, $|FC| \geq 1.5$; n=5). **b**, Heatmap of cardiac differentially expressed phosphopeptides identified in (**a**). For the heatmap, phosphopeptides were first sorted from most up-regulated to most down-regulated in KI:KO hearts compared to WT prior to unsupervised sample clustering using Euclidean distance measure and average linkage. **c**, Principal component analysis (PCA) of the total list of cardiac phosphopeptides identified in Fig. 2a found significantly altered across the mouse models vs WT (limma, $p < 0.05$, $|FC| \geq 1.5$; n=5). **d**, Consolidated phosphomotifs generated by iceLogo[69] of pSer- and pThr-modified phosphopeptides found differentially expressed in the mouse models compared to WT showing site-specific amino acid preferences adjacent to the central phosphorylated residue. **e**, Phosphopeptide sequence motif discovery by the MoMo[24] software tool based on the Motif-x[25] algorithm using the list of differentially expressed phosphopeptides found in each mouse model vs WT as input. **f**, Top 20 over-represented KEA2-determined biological terms in the altered mouse cardiac protein phosphosites. **g**,**h**, Top 10 significantly over-represented GO cellular component enriched terms (**g**) and IPA Canonical Pathways (**h**) among the altered cardiac DEPPs in each model vs WT. Terms are ordered by descending significance in the KI:KO model vs WT alongside a heatmap showing the number of associated DEPPs. See also Fig. 2.

Supplementary Figure 3

a

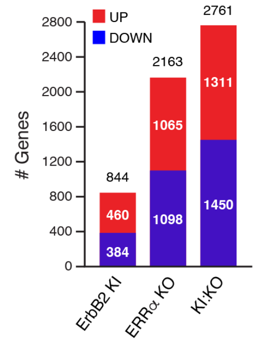

b

IPA: Selected Cardiac-related Toxicological Functions

	ErbB2 KI vs WT		ERRα KO vs WT		KI:KO vs WT	
Cardiac disease/function	***p-value***	***# genes***	***p-value***	***# genes***	***p-value***	***# genes***
Hypertrophy of heart	6.13E-04	29	3.21E-13	103	1.20E-11	121
Dilated cardiomyopathy	ns	13	3.65E-10	76	5.85E-10	92
Arrhythmia	1.64E-02	18	8.16E-11	74	2.71E-11	91
Myocardial infarction	5.01E-03	18	3.90E-09	64	1.39E-09	79
Left ventricular dysfunction	ns	12	3.55E-11	64	5.90E-10	74
Failure of heart	ns	9	1.33E-05	62	3.92E-04	71
Fibrosis of heart	ns	9	1.17E-04	49	1.80E-03	56
Coronary artery disease	ns	9	1.10E-03	41	1.27E-03	51
Enlargement of heart cells	2.38E-04	16	6.43E-07	42	7.50E-05	45
Congestive heart failure	ns	3	3.10E-05	32	1.32E-03	34
Damage of heart	ns	3	2.54E-06	22	2.26E-04	22

c

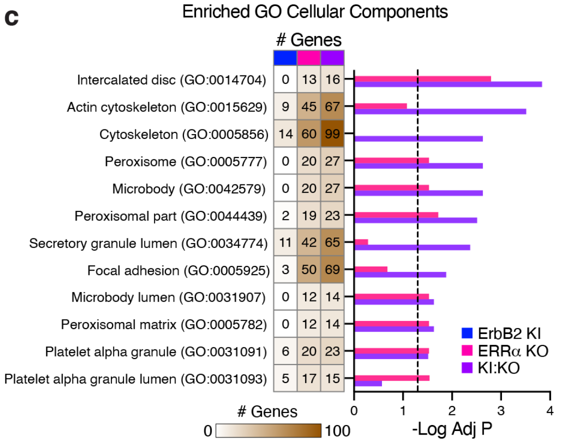

d

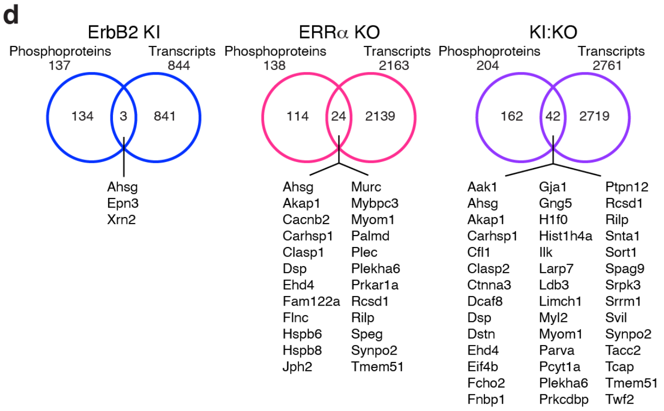

e

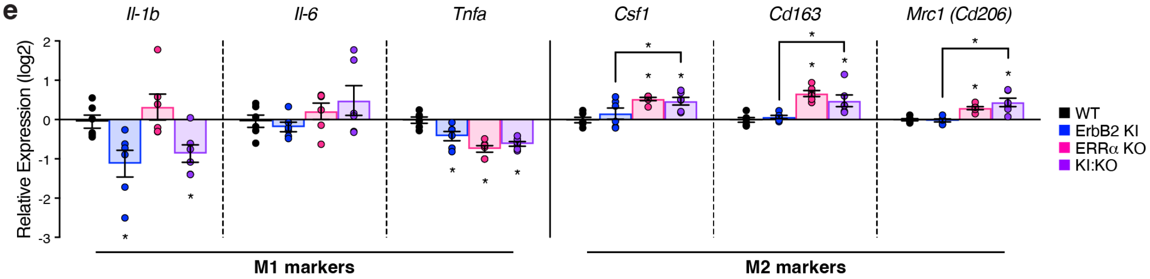

Supplementary Fig. 3 | Gene expression profiling of mouse hearts. a, Bar chart showing the number of up- and down-regulated cardiac DEGs identified by microarray analysis in the mouse models versus WT (ANOVA, $p < 0.05$, $|FC| \geq 1.2$; n=3). **b**, Enrichment of selected cardiac disease/functions identified by IPA analysis in mouse heart transcriptomes from (**a**). Terms are ordered by descending number of DEGs in the KI:KO model vs WT. ns: not significant ($p > 0.05$). **c**, Significant GO cellular component enriched terms in the cardiac DEGs identified in (**a**). Terms are ordered by descending significance in the KI:KO model vs WT alongside a heatmap showing the number of associated DEGs. No terms were significantly enriched in ErbB2 KI hearts. **d**, Overlap of cardiac DEPPs and DEGs identified by phosphoproteomics (n=5) and transcriptomics (n=3) experiments, respectively. **e**, qRT-PCR analysis of M1 and M2 macrophage gene markers in mouse hearts (n=6). Data are normalized to *Rplp0* levels. Data represent means ±SEM, *$p < 0.05$ by ANOVA relative to WT controls, unless otherwise indicated. See also Fig. 3.

Supplementary Figure 4

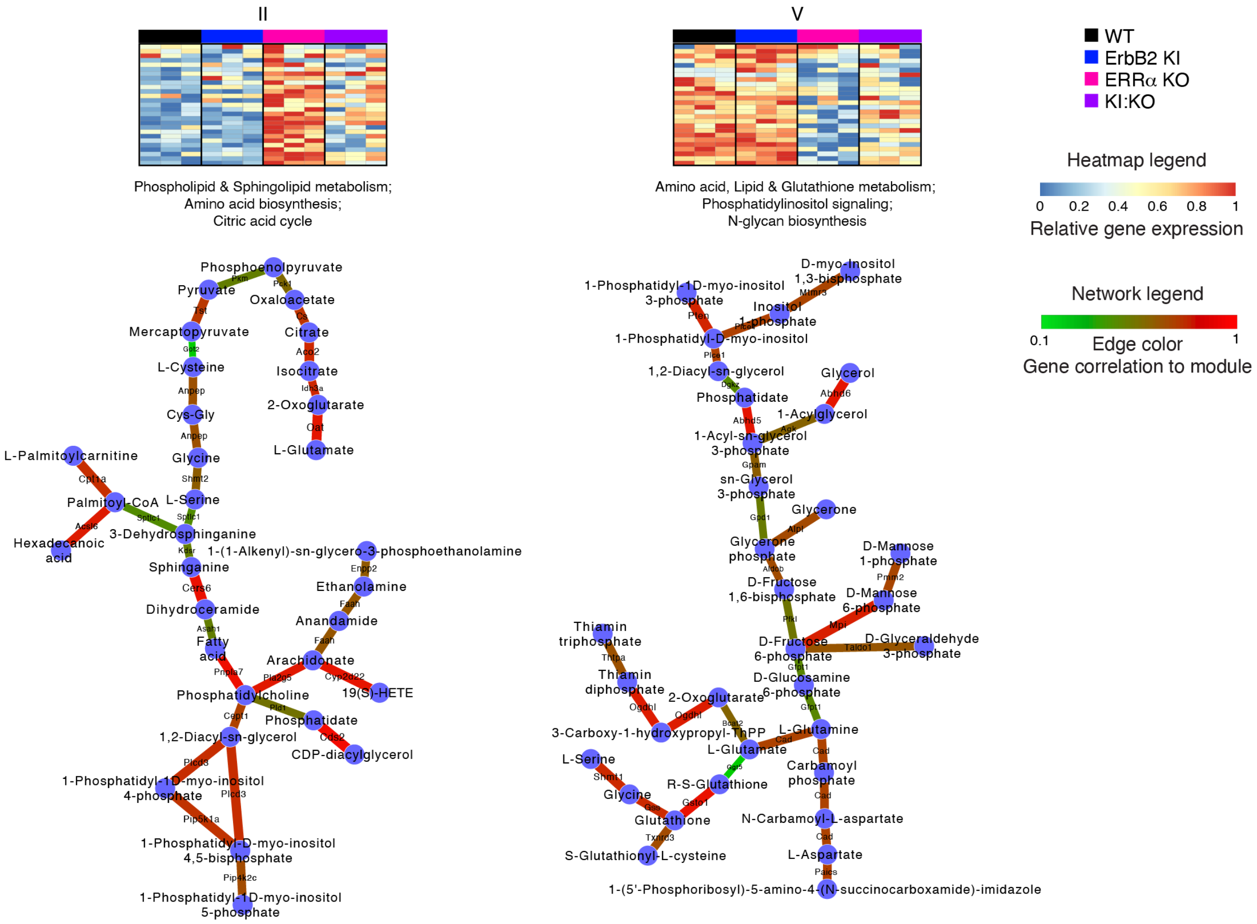

Supplementary Fig. 4 | Altered mouse cardiac metabolic modules identified from integrating transcriptomics data into metabolic networks. Computational integration of gene-level datasets with metabolic networks uncovered 7 significantly perturbed metabolic modules with the gene signature heatmap and network along with key enriched metabolic pathways for 2 of these metabolic modules shown. See also Fig. 4.

Supplementary Figure 5

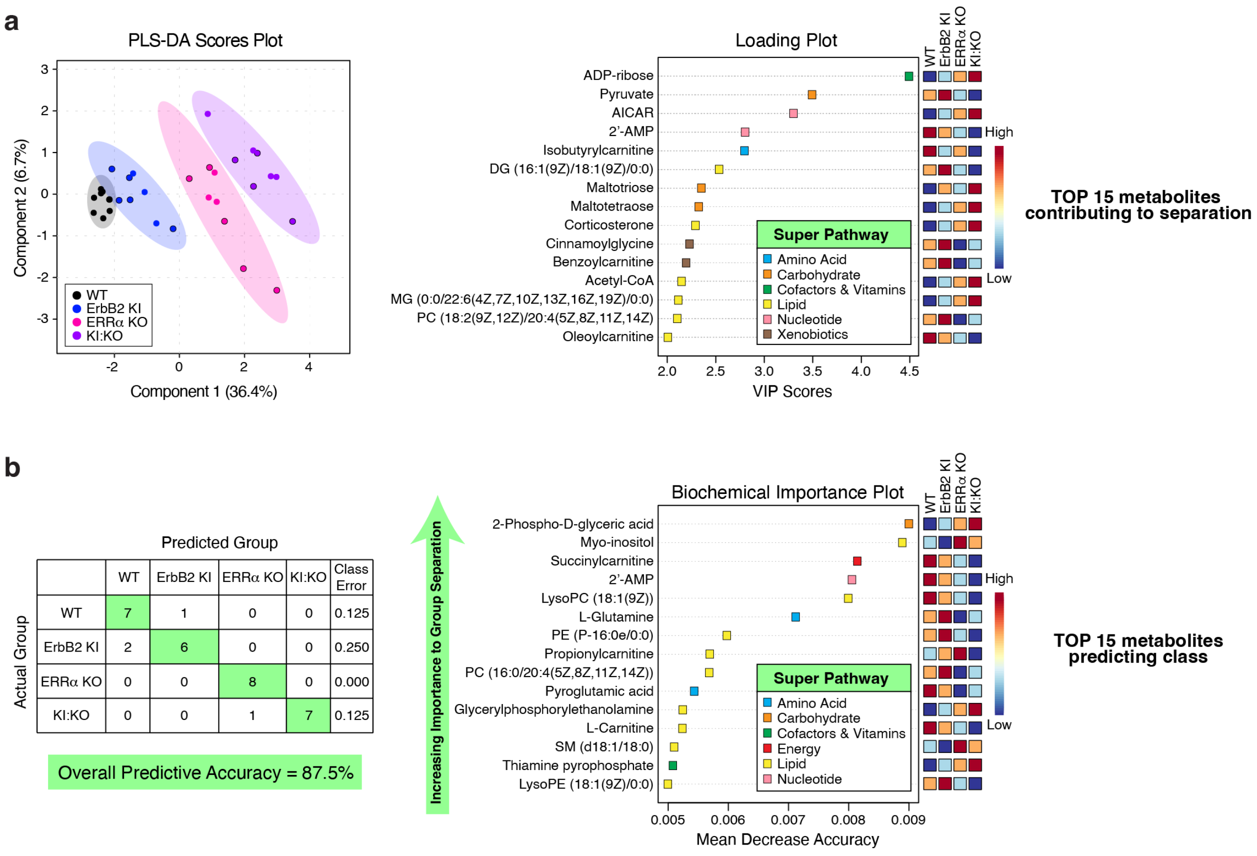

	WT	ErbB2 KI	ERRα KO	KI:KO	Class Error
WT	7	1	0	0	0.125
ErbB2 KI	2	6	0	0	0.250
ERRα KO	0	0	8	0	0.000
KI:KO	0	0	1	7	0.125

Supplementary Fig. 5 | Statistical classification of mouse cardiac metabolomes. a, Scores plot separation of genotypes based on the mouse heart metabolomes (n=8) using the supervised classification method partial least squares–discriminant analysis (PLS-DA). Loading plot of the top 15 metabolites contributing to the mouse group separation ranked by Variable Importance in Projection (VIP) scores is also shown. **b**, Random forest analysis of the mouse heart metabolomes (n=8) classified the genotypes with an overall predictive accuracy of 88%. Biochemical importance plot of the top 15 metabolites owing to the classification shows key differences in lipids. See also Fig. 5.

Supplementary Figure 6

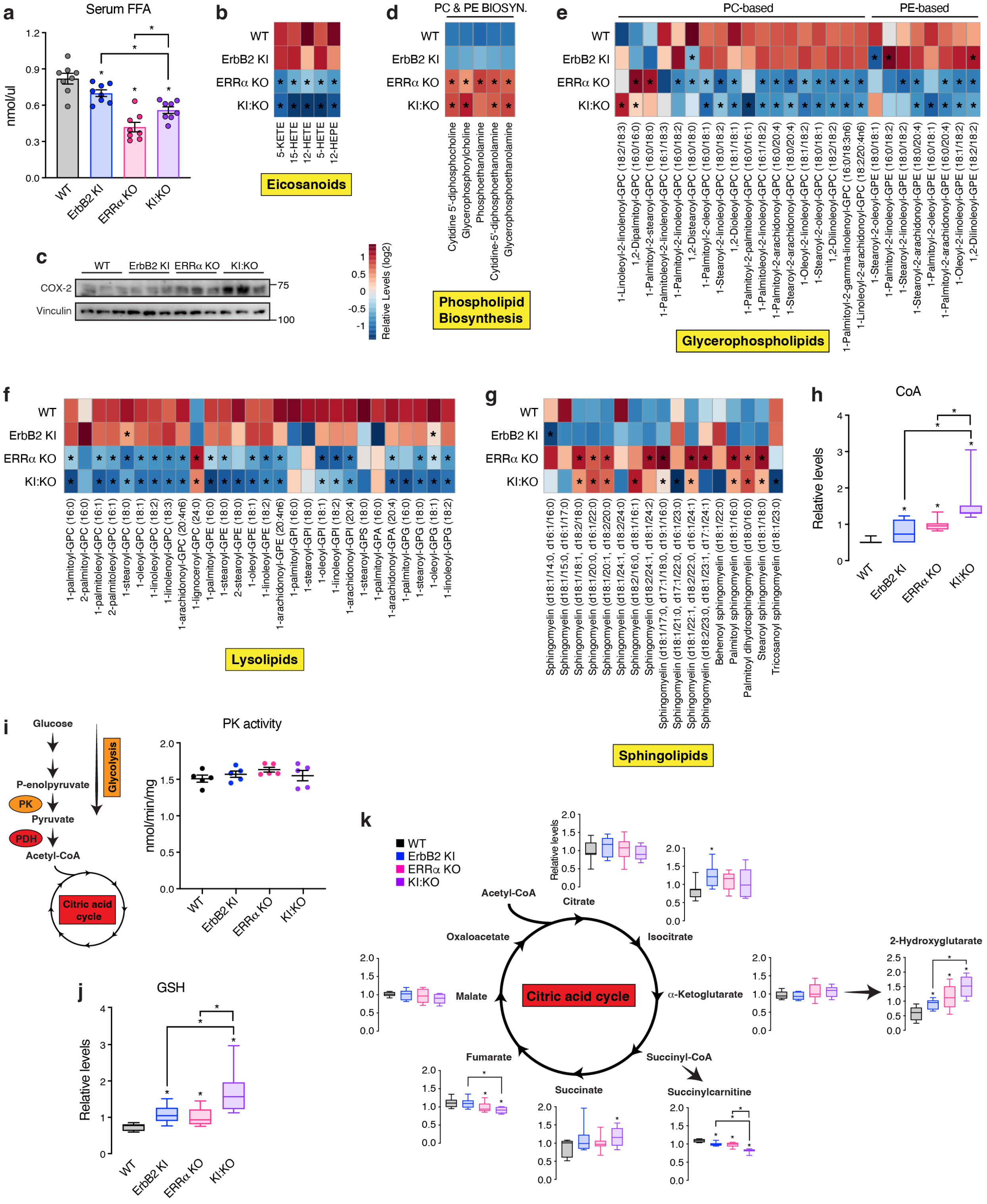

Supplementary Fig. 6 | Cardiac metabolomics reveals perturbations in membrane lipid composition, citric acid cycle, and glutathione levels. a, Bar chart of circulating free fatty acid levels in the mice (n=8). **b**, Heatmap of the expression profiles of eicosanoids in the mouse hearts (n=8). **c**, Immunoblot analysis of COX-2 in heart tissue extracts (n=3). Vinculin levels are shown as a loading control. **d-g**, Heatmap of the expression profiles of precursors in the biosynthesis of PC and PE phospholipids (**d**), glycerophospholipids (**e**), lysolipids (**f**) and sphingomyelin (**g**) lipid species in the mouse hearts (n=8). **h**, Box plot of mouse cardiac CoA levels (n=8). **i**, Schematic of pyruvate metabolism and scatter dot plot of mouse cardiac pyruvate kinase (PK) activities (n=5). **j**, Box plot of mouse cardiac glutathione (GSH, reduced) levels (n=8). **k**, Schematic of the citric acid cycle with box plots showing levels of intermediates and derivatives (n=8). Data in **h**, **j**, and **k** are shown as box and whiskers plots: center line denotes median, box extends from 25th to 75th percentiles, and whiskers extend to the lowest and highest values; *$p < 0.05$ by ANOVA relative to WT controls, unless otherwise indicated. Data in **a** and **i** represent means ±SEM; *$p < 0.05$ by ANOVA relative to WT controls, unless otherwise indicated. Metabolites with an asterix (*) in the heatmap representations (**b**, **d-g**) were found significantly altered in the indicated mouse model versus WT; *$p < 0.05$ by ANOVA. See also Fig. 5.

Supplementary Figure 7

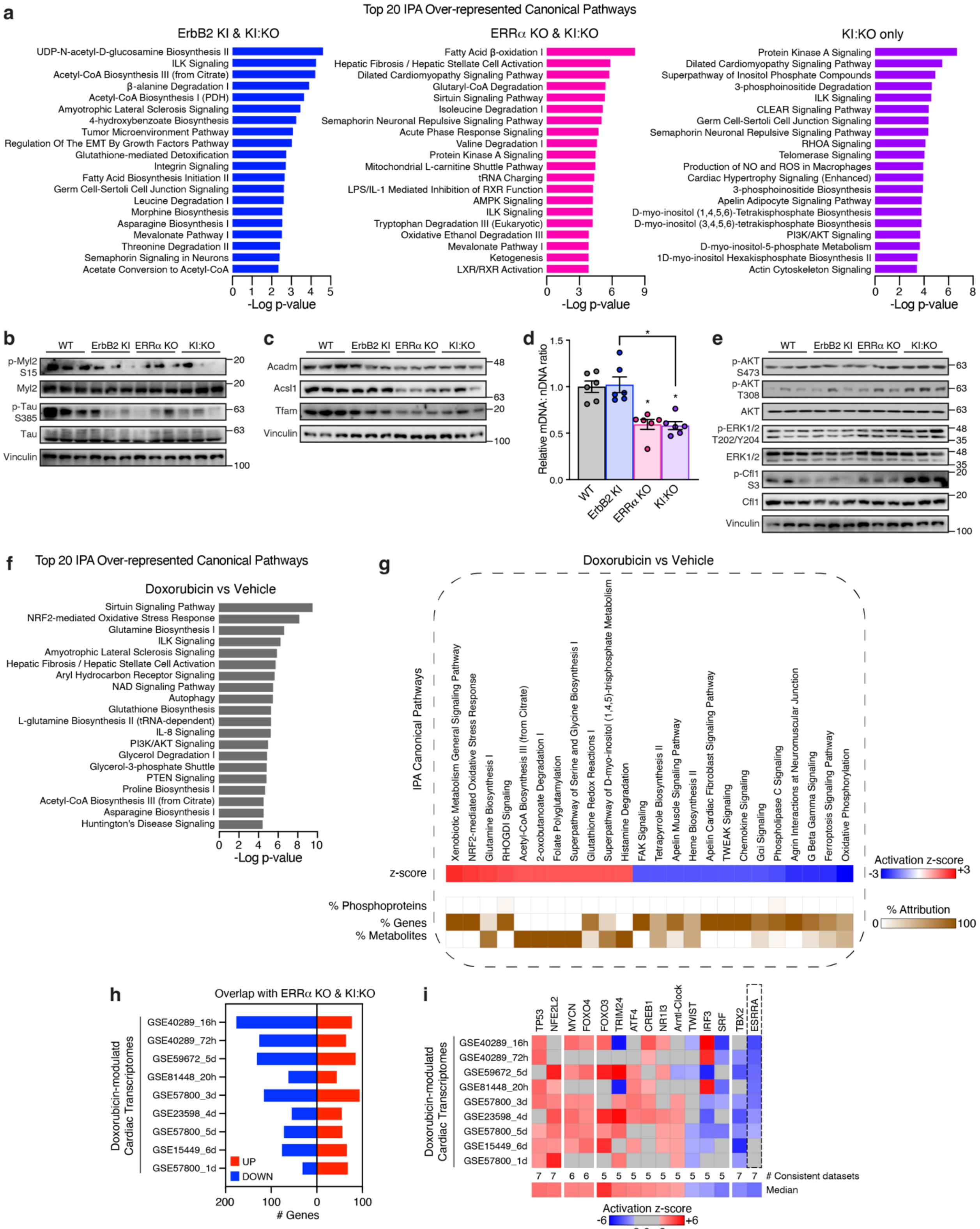

Supplementary Fig. 7 | Characterization of a cardiac doxorubicin multi-omics signature. a, Top 20 over-represented IPA-determined canonical pathways in the multi-omics signatures identified in Fig. 6a. **b**,**c**, Immunoblot analysis of phospho-Myl2 S15 and phospho-Tau S385 (**b**) as well as mitochondrial function-related proteins (**c**) in heart tissue extracts (n=3). Vinculin levels are shown as a loading control. **d**, Relative mouse cardiac mitochondrial to nuclear DNA (mtDNA:nDNA) ratios (n=6). **e**, Immunoblot analysis of AKT and ERK1/2 signaling activities and phospho-Cfl1 S3 levels in heart tissue extracts (n=3). Vinculin levels are shown as a loading control. **f**,**g**, IPA canonical pathway enrichment (**f**) and activity (**g**) analyses of the constructed multi-omics cardiac doxorubicin signature identified in Fig. 6d. Pathways with significantly associated activation (z-score ≥ 2) or inhibition (z-score $\leq$-2) states are shown with the relative contribution of each omics layer (phosphoprotein, gene, metabolite) to the predictions. **h**, Number of up- and down-regulated genes commonly deregulated in ERRα KO and KI:KO hearts that were also found similarly deregulated in 9 independent cardiac doxorubicin-modulated transcriptomes. **i**, IPA upstream regulator analysis of 9 independent cardiac doxorubicin-modulated transcriptomes. Transcriptional regulators/nuclear receptors associated with significant activation (z-score ≥ 2) or inhibition (z-score $\leq$-2) states are shown and organized by both their degree of consistency across the datasets and median activation score. Data in **d** represent means ±SEM; *$p < 0.05$ by ANOVA relative to WT controls, unless otherwise indicated. See also Fig. 6.

Supplementary Figure 8

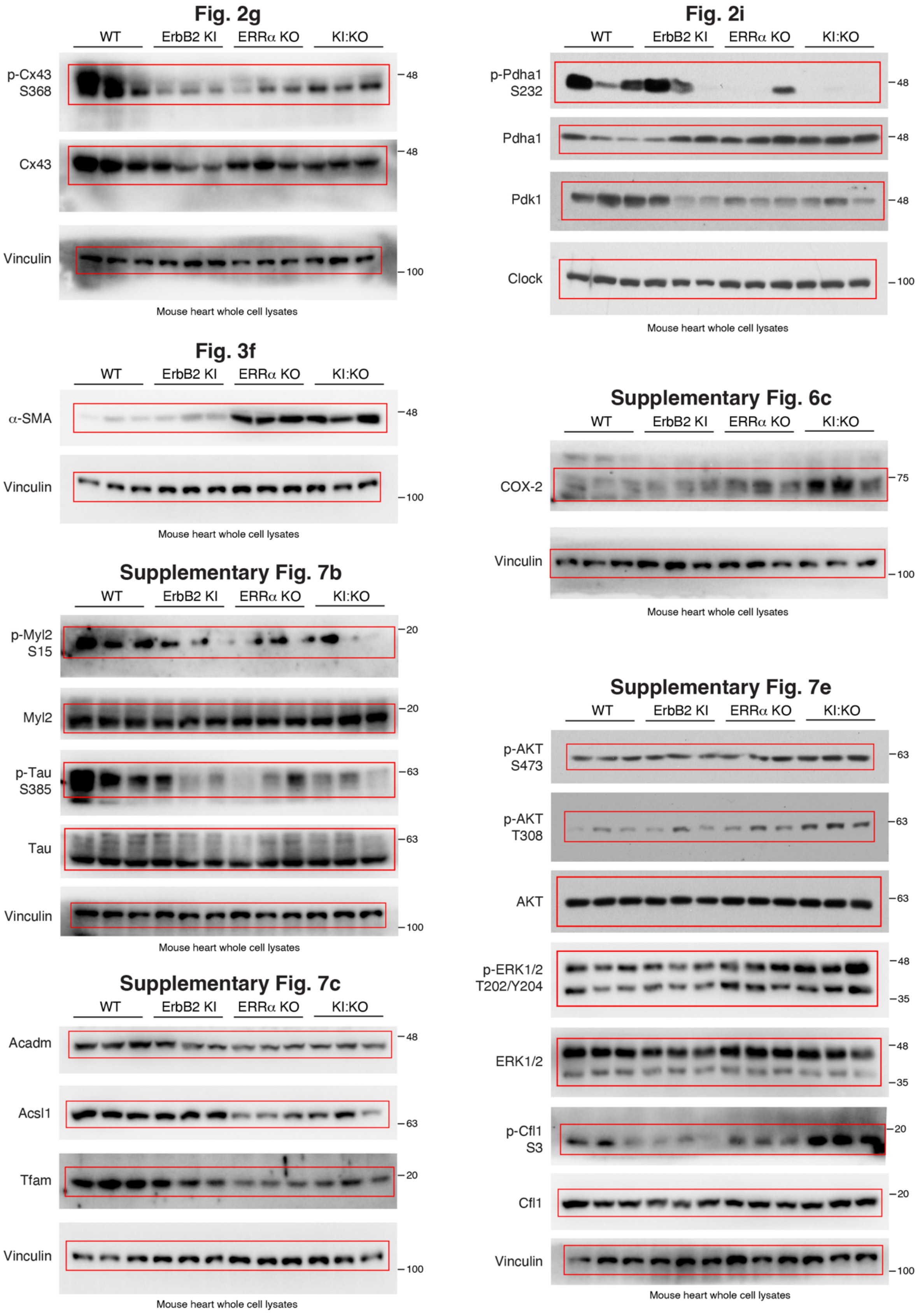

Supplementary Fig. 8 | Uncropped immunoblots. Western blot membranes were generally sliced to maximize sample use by increasing the number of protein detections per gel. Portions of the blots used to generate figures are indicated by red boxes. Related to Fig. 2g,i, 3f, and Supplementary Fig. 6c, 7b,c,e.
