# Supplementary material for: Integrated multi-omics analysis of adverse cardiac remodeling and metabolic inflexibility upon ErbB2 and ERRα deficiency
Source: Commun Biol. 2022 Sep 12;5:955. doi: 10.1038/s42003-022-03942-4 (PMC9467976; doi:10.1038/s42003-022-03942-4)
Supplement: Supplementary file 3 — Description of Additional Supplementary Files [file 42003_2022_3942_MOESM3_ESM.pdf]

Description of Additional Supplementary Files

File name: Supplementary Data 1
Description: Phosphoproteomics profiling and functional analysis of ErbB2 KI, ERRα KO and KI:KO mouse hearts versus WT (related to Figures 2 and S2).

File name: Supplementary Data 2
Description: Transcriptomics profiling and functional analysis of ErbB2 KI, ERRα KO and KI:KO mouse hearts versus WT (related to Figures 3, 4, S3, and S4).

File name: Supplementary Data 3
Description: Metabolomics profiling of ErbB2 KI, ERRα KO and KI:KO mouse hearts versus WT (related to Figures 5, S5, and S6).

File name: Supplementary Data 4
Description: ErbB2- and/or ERRα- dependent integrated omics signatures with increased likelihood for DCM disease causality in KI:KO mouse hearts and associated perturbed IPA canonical pathways (related to Figures 6 and S7).

File name: Supplementary Data 5
Description: Functional and comparative analysis of an assembled cardiac doxorubicin multi-omics signature with the ERRα & KI:KO multi-omics signature (related to Figures 6 and S7).

File name: Supplementary Data 6
Description: Mouse primers for mitochondrial content determination and RTqPCR (related to Figures 1, 3, 5, S1, S3, and S7).

File name: Supplementary Data 7
Description: Source data underlying the graphs (related to Figures 1, 2, 3, 5, S1, S3, S6, and S7).
