# Supplementary material for: Integrated multi-omics analysis of adverse cardiac remodeling and metabolic inflexibility upon ErbB2 and ERRα deficiency
Source: Commun Biol. 2022 Sep 12;5:955. doi: 10.1038/s42003-022-03942-4 (PMC9467976; doi:10.1038/s42003-022-03942-4)
Supplement: Supplementary file 11 — Reporting Summary [file 42003_2022_3942_MOESM11_ESM.pdf]

nature portfolio

Corresponding author(s): Vincent Giguère

Last updated by author(s): Aug 25, 2022

Reporting Summary

Nature Portfolio wishes to improve the reproducibility of the work that we publish. This form provides structure for consistency and transparency in reporting. For further information on Nature Portfolio policies, see our Editorial Policies and the Editorial Policy Checklist.

Statistics

For all statistical analyses, confirm that the following items are present in the figure legend, table legend, main text, or Methods section.

n/a	Confirmed	
☐	☒	The exact sample size (*n*) for each experimental group/condition, given as a discrete number and unit of measurement
☐	☒	A statement on whether measurements were taken from distinct samples or whether the same sample was measured repeatedly
☐	☒	The statistical test(s) used AND whether they are one- or two-sided<br>*Only common tests should be described solely by name; describe more complex techniques in the Methods section.*
☒	☐	A description of all covariates tested
☐	☒	A description of any assumptions or corrections, such as tests of normality and adjustment for multiple comparisons
☐	☒	A full description of the statistical parameters including central tendency (e.g. means) or other basic estimates (e.g. regression coefficient) AND variation (e.g. standard deviation) or associated estimates of uncertainty (e.g. confidence intervals)
☐	☒	For null hypothesis testing, the test statistic (e.g. *F*, *t*, *r*) with confidence intervals, effect sizes, degrees of freedom and *P* value noted<br>*Give P values as exact values whenever suitable.*
☒	☐	For Bayesian analysis, information on the choice of priors and Markov chain Monte Carlo settings
☒	☐	For hierarchical and complex designs, identification of the appropriate level for tests and full reporting of outcomes
☒	☐	Estimates of effect sizes (e.g. Cohen's *d*, Pearson's *r*), indicating how they were calculated

Our web collection on statistics for biologists contains articles on many of the points above.

Software and code

Policy information about availability of computer code

Data collection

-Vevo 2100 (VisualSonics) for echocardiography.
-Zeiss Axio Scan.Z1 instrument for IF images.
-Aperio ScanScope instrument for H&E, Masson's trichrome, TUNEL assay, and IHC images.
-Epson Perfection V700 Photo or Bio-Rad's ChemiDoc MP imaging System for immunoblot images.
-Roche LightCycler 480 instrument for RT-qPCR.
-MaxQuant (v 1.6.1.0) and Perseus (v 1.6.0.7) software for phosphoproteomics analysis.

Data analysis

-Vevo 2100 (VisualSonics) for echocardiography.
-Fiji software for IF analysis.
-Aperio's ImageScope software for histological and IHC analyses.
-HALO software (Indica Labs) for detection of TUNEL-positive cells.
-GraphPad Prism 9 for statistics and graphing purposes.
-Limma R/bioconductor package (PMID: 25605792) for phosphoproteomics statistical analysis.
-Morpheus (https://software.broadinstitute.org/morpheus/) for heatmap generation.
-Phantasus v 1.11.0 (https://artyomovlab.wustl.edu/phantasus/) for principal component analysis (PCA).
-The algorithms PHOSIDA, iceLogo, and MoMo (v 5.4.1; based on Motif-x) for phosphopeptide consensus motifs.
-PhosphoMotif Finder tool (http://www.hprd.org/PhosphoMotif_finder) for kinase prediction of phosphomotifs.
-KEA2 (https://www.maayanlab.net/KEA2/) for enriched biological terms of altered phosphosites.
-Ingenuity Pathway Analysis (IPA, Qiagen, v Spring release 2022) for enriched and/or modulated canonical pathways, cardiac-related toxicological functions, and upstream regulator analysis.
-Enrichr (https://maayanlab.cloud/Enrichr/) for enriched GO cellular component analysis (2018).
-GSEA (https://www.gsea-msigdb.org/gsea/index.jsp) for enriched Molecular Signature Database (MSigDB, v 5.2) hallmark signatures.
-Expression Console (v 1.4.1) and Transcriptome Analysis Console (v 3.0) software (Affymetrix, Inc.) were used for microarray analysis.
-The transcriptome-based metabolic network clustering analysis was done as described in PMID: 27771149 and https://

doi.org/10.1101/2020.07.15.204388).
-Metabolon's hardware and software for untargeted metabolomics data analysis.
-MetaboAnalyst (v 5.0) for heatmap, PLD-DA, and Random forest classification of groups based on metabolites.

For manuscripts utilizing custom algorithms or software that are central to the research but not yet described in published literature, software must be made available to editors and reviewers. We strongly encourage code deposition in a community repository (e.g. GitHub). See the Nature Portfolio guidelines for submitting code & software for further information.

Data

Policy information about availability of data

All manuscripts must include a data availability statement. This statement should provide the following information, where applicable:

- Accession codes, unique identifiers, or web links for publicly available datasets
- A description of any restrictions on data availability
- For clinical datasets or third party data, please ensure that the statement adheres to our policy

Phosphoproteomics data have been deposited to the ProteomeXchange Consortium via the PRIDE partner repository with the dataset identifier PXD032766. Microarray data have been deposited in the NCBI Gene Expression Omnibus (GEO) under accession number GSE199150. Metabolomics data have been deposited to the EMBL-EBI metabolomics repository, MetaboLights, with the dataset identifier MTBLS795. All omics datasets involving doxorubicin studies in mice or rats analyzed in the current study are publicly available and summarized in Supplementary Table 5.

Field-specific reporting

Please select the one below that is the best fit for your research. If you are not sure, read the appropriate sections before making your selection.

☒ Life sciences ☐ Behavioural & social sciences ☐ Ecological, evolutionary & environmental sciences

For a reference copy of the document with all sections, see nature.com/documents/nr-reporting-summary-flat.pdf

Life sciences study design

All studies must disclose on these points even when the disclosure is negative.

Sample size	Sample sizes for each experiment are indicated and were based on previous reports in the literature with statistical analyses validating the significance of the findings.
Data exclusions	N/A
Replication	Experimental design was based on the use of at least 3 biological replicates as described in the figure legends and methods section generating consistent data as indicated by the significance of the results.
Randomization	Mice from each genetic model were age- and sex-matched. Body weights were not matched given the influence of the genetic model on body weight.
Blinding	For blood/tissue collection from mice, animals were not blinded as mice were sacrificed in rotation between mouse models to minimize potential circadian effects. Omics data collection were performed in a blinded manner. Data analyses were performed by different individuals.

Reporting for specific materials, systems and methods

We require information from authors about some types of materials, experimental systems and methods used in many studies. Here, indicate whether each material, system or method listed is relevant to your study. If you are not sure if a list item applies to your research, read the appropriate section before selecting a response.

Materials & experimental systems

n/a	Involved in the study	
☐	☒	Antibodies
☒	☐	Eukaryotic cell lines
☒	☐	Palaeontology and archaeology
☐	☒	Animals and other organisms
☒	☐	Human research participants
☒	☐	Clinical data
☒	☐	Dual use research of concern

Methods

n/a	Involved in the study	
☒	☐	ChIP-seq
☒	☐	Flow cytometry
☒	☐	MRI-based neuroimaging

Antibodies

Antibodies used	-anti-rabbit (GE Healthcare, Cat# NA9340, Research Resource Identifier (RRID):AB_772191).<br>-anti-mouse (GE Healthcare, Cat# NA934, RRID:AB_772206).<br>-anti-goat (Santa Cruz Biotechnology, Cat# sc-2020, RRID:AB_631728).<br>-phospho-Cx43 Ser368 (Cell Signaling Technology, Cat# 3511, RRID:AB_211016).<br>-Cx43 (Millipore, Cat# 3512, RRID:AB_229459).<br>-Vinculin (Santa Cruz Biotechnology, sc-25336, RRID:AB_628438).<br>-phospho-Pdha1 Ser232 (Millipore, Cat# AP1063, RRID:AB_10616070).<br>-Pdha1 (Cell Signaling, Cat# 3205, RRID:AB_2162926).<br>-Pdk1 (Enzo Life Sciences, Cat# ADI-KAP-PK112, RRID:AB_10618932).<br>-Clock (Santa Cruz Biotechnology, Cat# sc-6927, RRID:AB_2082577).<br>-α-SMA (Thermo Fisher Scientific, Cat# 14-9760-82, RRID:AB_2572996).<br>-COX-2 (Cell Signaling Technology, Cat# 12282, RRID:AB_2571729).<br>-phospho-Myl2 Ser15 (Thermo Fisher Scientific, Cat# PA5-104265, RRID:AB_2816014).<br>-Myl2 (Cell Signaling Technology, Cat# 3672, RRID:AB_10692513).<br>-phospho-Tau Ser396 (Ser385 in mouse) (Abclonal, Cat# AP1028, RRID:AB_2863912).<br>-Tau (Abclonal, Cat# A0002, RRID:AB_2756869).<br>-Acadm (St John's Laboratory, Cat# STJ96389, RRID:AB_2922676).<br>-Acsl1 (Cell Signaling Technology, Cat# 4047, RRID:AB_2222411).<br>-Tfam (Abcam, Cat# ab131607, RRID:AB_11154693).<br>-phospho-AKT Ser473 (Cell Signaling Technology, Cat# 9271, RRID:AB_329825).<br>-phospho-AKT Thr308 (Cell Signaling Technology, Cat# 9275, RRID:AB_329828).<br>-AKT (Cell Signaling Technology, Cat# 9272, RRID:AB_329827).<br>-phospho-ERK1/2 (p44/42 MAPK) T202/Y204 (Cell Signaling Technology, Cat# 9101, RRID:AB_331646).<br>-ERK1/2 (p44/42 MAPK) (Cell Signaling Technology, Cat# 9102, RRID:AB_330744).<br>-phospho-Cfl1 S3 (Cell Signaling Technology, Cat# 3313, RRID:AB_2080597).<br>-Cfl1 (Abclonal, Cat# A2658, RRID:AB_2922675).<br>-Aif-1 (Iba-1, FUJIFILM Wako Pure Chemical Corporation, Cat# 019-19741, RRID:AB_839504) for IHC.<br>-CD31 (PECAM-1, Cell Signaling, Cat# 77699, RRID:AB_2722705) for IHC.<br>-α-SMA (alpha-smooth muscle actin, Thermo Fisher Scientific, Cat# 14-9760-82, RRID:AB_2572996) for IHC.<br>-Wheat germ agglutinin (WGA) conjugated to Alexa Fluor 488 (Invitrogen, W11261) for IF.
Validation	All antibodies were commercially available and validated by the manufacturer. The antibodies were all used in other publications.

Animals and other organisms

Policy information about studies involving animals; ARRIVE guidelines recommended for reporting animal research

Laboratory animals	In this study, male mice aged 15 weeks of age in an FVB genetic background were used. The genetic mouse models used are: WT, ERRα KO (PMID:14585956), ErbB2 cDNA knock-in (Erbb2 KI; PMID:15496447), and KI:KO mice generated herein from breeding ERRα KO mice with Erbb2 KI mice.
Wild animals	N/A
Field-collected samples	N/A
Ethics oversight	All animal experimentations were conducted in accord with accepted standards of humane animal care and all protocols were approved by the McGill Facility Animal Care Committee and the Canadian Council on Animal Care.

Note that full information on the approval of the study protocol must also be provided in the manuscript.
